# Microstructure and Degradation of Mortar Containing Waste Glass Aggregate as Evaluated by Various Microscopic Techniques

**DOI:** 10.3390/ma13092186

**Published:** 2020-05-09

**Authors:** Przemysław Czapik

**Affiliations:** Department of Building Engineering Technologies and Organization, Kielce University of Technology, Al. Tysiąclecia Państwa Polskiego 7, 25-314 Kielce, Poland; p.czapik@tu.kielce.pl

**Keywords:** alkali-silica reaction (ASR), waste glass aggregate, mortar degradation, optical microscopy, SEM-EDS, backscattered electron imaging

## Abstract

The primary aim of this article is to focus on the alkali-silica reaction (ASR) in mortar specimens containing coloured waste glass used as an aggregate. Mortar expansion was measured using the ASTM C 1260 accelerated test procedure until the specimens disintegrated. Special attention was paid to the microscopic examination of the damaged mortar. Various methods were used for this purpose, including optical microscopy in reflected and transmitted light with one and two crossed polarizers. The specimens were also subjected to the scanning electron microscopy observations with energy dispersive spectroscopy (SEM-EDS). The data obtained from these techniques provided information on the mechanism of glass-containing mortar degradation due to ASR and also allowed the comparison of different microscopic techniques in terms of the information they can provide on ASR occurrence.

## 1. Introduction

The alkali-silica reaction (ASR) is a concrete degradation phenomena, in which sodium and potassium hydroxides react with the aggregate containing reactive forms of silica [1,2,3]. The reactive silica in the aggregate occurs in amorphous or metastable crystalline forms, such as cristobalite, tridymite, and moganite in addition to strained quartz, chalcedony and amorphous silica [1,2]. Aggregates that contain amorphous silica exhibit the highest reactivity level [3,4]. In view of that, the borosilicate glass is used as a reference aggregate for tests [5,6]. In research practice, the opal aggregate composed of amorphous silica [7,8,9,10] is used more often than other, less reactive, types of aggregate to investigate concrete degradation due to ASR [11,12].

Despite potentially high reactivity of glass, it is used for the production of concrete, both as a reinforcement in the form of glass fibres [13,14] and as a lightweight aggregate in the form of foam glass [15,16]. Another important issue is the potential use of waste glass (WG) for the production of concrete. In theory, glass should be in 100% recyclable, however in Poland a large amount of used glass containers and other waste glass goes to landfills, which is disadvantageous both economically and environmentally [17]. The increase in reclaimed glass implementation in concrete production is a goal of intensive studies [18,19,20,21,22,23,24,25]. As glass is composed of amorphous silica, the finely ground glass powder has pozzolanic properties and can be used as a mineral additive to cement [18,19,20]. For the same reason, the use of waste glass as a substitute for aggregate may expose concrete to ASR-induced degradation [6,19,20,21,22,25,26,27,28,29,30]. This is because waste glass, unlike glass fibres deliberately manufactured for use in concrete, is not coated to counteract ASR [31]. Degradation of the cement composite with waste glass is not the rule, although the results of standard expansion tests often indicate an increased reaction rate in the final test periods, even when the ultimate test result is that the specimen under examination exhibits no deleterious reaction [22,25]. Waste glass fineness is of great importance here [32]. From a chemical point of view, ASR is very similar to s pozzolanic reaction [19,33,34,35]. Waste glass in concrete can both cause and inhibit alkali-silica reaction [20,32,36]. Thus, when waste glass is added to the concrete, the ASR-induced degradation of larger grains may be limited by smaller ones entering into pozzolanic reaction. According to the studies by Hou et al. [34], products resulting from these two reactions differ in the degree of polymerization. As a result of ASR, more polymerized products are formed, which, by using calcium and reactive silica from the environment, take on the features of a gel. Thus, as reported by Moon et al. [35], the alkaline gel has a lower density and higher compressibility than amorphous silica, from which it can be formed.

According to Matos and Sousa-Coutinho [18], pozzolanic properties may be exhibited by a glass particle less than 0.3 mm in size, being particularly pronounced when it is less than 0.1 mm. Shayan and Xu [20] claim that to mitigate ASR with glass as a pozzolanic additive, the glass particle must be no more than 0.15 mm, although other tests indicate a particle size of less than 0.075 mm. Shao et al. [25] argue that glass only becomes pozzolanic when ground to a grain size below 0.038 mm. Idir et al. [19] remark that a slight but significant pozzolanic activity may be exhibited by glass grains larger than 140 μm. However, the authors indicate here that this can expose concrete to ASR degradation. The products of alkali-silica reaction were detected for glass with a grain size of more than 1.25 mm in scanning electron microscopy (SEM) and X-ray fluorescence (XRF) tests. The findings of those phase composition studies are consistent with the results of expansion tests conducted for composites with waste glass aggregates [19,27,28,36]. Various studies indicate that the minimum size of glass grains that may cause concrete degradation due to ASR ranges from 0.5 [27,36] to 1.25 mm [19]. Nevertheless, susceptibility of aggregate to ASR does not increase linearly with increasing grain size. There is some pessimum from which a decrease in aggregate reactivity resulting from the increase in grain size is observed. Therefore, in order to quickly obtain clear results of cement composite degradation due to ASR, sand (fractions up to 5 mm) is usually used as an aggregate [37,38]. In the case of glass, the pessimum granulation for which the degradation due to ASR is the greatest has been determined at the level of 1.2 mm [20] or 1.8 mm [36]. Differences in test results may arise from many factors. In addition to glass grain size, the type of glass (chemical composition), texture, its volume content in the sample, etc. play a role [12,27,36]. It should also be noted that very fine fractions of pozzolanic materials may agglomerate and lead to alkali-silica reaction [39].

Microstructural observations often complement the evaluation of ASR progress through expansion measurements and usually correlate well with the results of the evaluation [19,36]. The greatest changes in the specimen volume are usually associated with the greatest changes in the microstructure, manifested by the formation of ASR products and crack networks. Usually a scanning electron microscope is used for microstructural testing. As for ASR, the tests are aimed at determining ASR products and the mechanism of their formation that contribute to the degradation of cement composite. The techniques may vary depending on the aggregate type and cement composite composition. Aggregate destruction is influenced by, among others, the type of reactive silica present, its shape and texture [12,36,40]. The formation of characteristic cracks [40] and other symptoms of aggregate degradation depend on these characteristics [7,41]. ASR products are initially formed on the surface of aggregate grains or in cracks occurring in them, i.e., in places where the pore solution rich in sodium and potassium has the easiest access to reactive silica [42]. The reaction proceeds towards the centre of reactive aggregate [43].

On the aggregate surface, a highly hydrated gel saturated with alkalis is formed, partially dissolved in the pore liquid, with the structure similar to that of water glass or sol [42,44]. Although the amorphous nature of ASR products is often assumed, there are studies demonstrating that the products of more orderly structure may be formed during the reaction [42,44]. One of the ASR theories assumes that, over time, the formed gel products may crystallize increasing in volume, which leads to concrete damage [45]. In the microstructure of concrete in which the alkali-silica reaction takes place, different products are identified [12,46]. They may take various forms of loose gels with a “house of cards” [27,47] structure or sword-like or rosette-like morphologies [48]. Such products can often be observed in the air voids in concrete with reactive aggregate. Due to usually limited space in which the ASR products can be formed, their formation in the form of a dense alkali gel is often observed [7,8,46]. These products cause stresses leading to cracks in the aggregate, which are then transferred to the surrounding paste [10,46,49]. The cracks, with widths of more than 10 μm, are usually wider than the voids between the grains and cracks formed earlier in the concrete. The cracks formed in the aggregate create an access path for the pore liquid to the unreacted aggregate, allowing ASR to continue its deleterious action in concrete. The subsequent ASR products can fill the cracks with widths from 10 to 100 μm.

Gao et al. [50] proposed an interesting model of ASR. The model assumes that the formation of gel in the initial stage is not exclusively a surface phenomenon. The gels also form just below the aggregate surface, which is usually associated with the presence of pores in the aggregate. In the case of waste glass, however, their role may be taken over by the residual cracks which appear during crushing and grinding. According to Maraghechi et al. [36], it is in such cracks having more than 2.5 μm in width and not on the grain surface that the alkali-silica reaction for glass begins. The ASR products which are formed inside the grains contribute to the propagation of these cracks and creation of new ones. Products that fill the cracks in the glass usually take the form of a compact alkali gel, sometimes with transverse cracks [6,51]. By reference to the studies conducted by Shin et al. [6], it can be concluded that these are crack-filling products of types 2 and 3. The cracks of less than 2.5 μm can thus be assigned to type 1 cracks. On the other hand, Boehm-Courjault et al. [42] found the presence of alkali-silica reaction products in the cracks with a width below 1 μm.

The microstructure of concrete and mortar with reactive aggregate can also be examined using microscopic techniques other than SEM [41]. Optical microscopy is usually used to examine aggregate alkali to determine the presence of reactive phases [37,47]. It is far less often used to assess the condition of cement composites in which alkali-silica reaction occurred [52,53]. However, in applications, such as examining cracks propagating in aggregates and cement paste, alkali gel, reactive rims around aggregate grains and the loss of the cement paste-aggregate bond, microscopic techniques will be a useful tool capable of characterizing the mechanism of ASR-induced deterioration of concrete [52].

Testing the waste glass susceptibility to ASR is based predominantly on the accelerated reactivity test [20,21,22,25,26,30], often supplemented with microstructure analysis using scanning electron microscopy. The aim of this study is to compare the information derived from various microscopic techniques used to evaluate the degradation of mortar containing waste glass as an aggregate. The outcome will allow investigating the waste glass degradation mechanism in a strongly alkaline cement paste environment. Bearing in mind the limitations of the accelerated ASTM C 1260 mortar expansion test method [38], the test period was extended from the standard 14 days to the moment of mortar specimen failure, when further measurements were impossible. This was done in order to obtain more evident results and be able to better describe the process of waste glass degradation in cement paste.

## 2. Materials and Methods

The tests of the susceptibility of waste glass to ASR were carried out on mortar specimens made and stored in accordance with ASTM C 1260 [38]. The specimens were made with CEM I 42.5R. All aggregate fractions were replaced with the corresponding size ranges of waste glass. The glass was derived from the broken and then ground brown bottles of the same type. After grinding in a ball mill and sieving to obtain the required fractions the glass was mixed according to ASTM C 1260 standard [38]. The chemical compositions of cement and glass determined with XRF (manufacturer Panalytical, Almelo, Netherlands) are shown in Table 1. The glass composition is indicative of the soda-lime type.

The mortar was prepared by mixing 420 g of cement and 945 g of aggregate in the form of ground waste glass with a gradation of 0.15 ÷ 4 mm and w/c ratio 0.47. Four 25 mm × 25 mm × 250 mm bars were made from that mortar. In compliance with the standard [30], after 1 day of curing in the moulds and subsequently after 1 day of storage at 80 °C in water, the mortar bars were moved to 1M NaOH solution at 80 °C and the expansion measurements were started. After 14 days of immersion in the NaOH solution, one bar was destroyed to obtain a fracture surface for observation with scanning electron microscopy (SEM) (FEI Company, Hillsboro, OR, USA). The other bars were left until ASR-induced disintegration.

One of the specimens damaged due to ASR was first examined visually. Subsequently, a polished section and a thin section were prepared. Due to the extent of damage induced by the specimen curing, the mortar bar was polymer-impregnated prior to the microscopic examination. The impregnation of the polished section was superficial to ensure that the specimen core microstructure remained unchanged. Before impregnation, the specimen cut from the mortar bar was dried by 2 h in a laboratory dryer. Epoxy resin was applied to impregnate the specimen.

Optical microscopy with reflected and transmitted light, both non-polarized and polarized, as well as SEM were applied for testing. Reflected light microscopy was used to investigate the fractured cracked surface of the ASR damaged bar and to examine the polished section. The thin section was examined in transmitted light and in the scanning electron microscope, in which the polished surface was also examined. The KEYENCE VHX 7000 microscope (KEYENCE, Osaka, Japan) was used for initial visual inspection of the fractured specimen. The reflected and transmitted light examinations were performed using an OLYMPUS BX51 microscope (Olympus, Tokyo, Japan). The SEM examination were performed with help of FEI Quanta FEG 250 microscope FEI Company, Hillsboro, OR, USA) equipped with a secondary electron (SE) detector, back-scattered electron (BSE) detector and energy dispersive X-ray microanalyzer (EDS). The microstructures of both the thin and the polished sections were examined under low vacuum conditions without sputtering pre-treatment, using 15-kV voltage.

## 3. Results and Discussion

The results of this study are presented in four separate subsections according to the technique used. First, the expansion test results for the mortar with waste glass are discussed and the evidence for and scale of ASR-induced damage are provided. The following two subsections describe the microstructure of degraded samples tested by different optical microscopy techniques. Next, the application of optical microscopy in reflected and transmitted light is described. Due to the large amount of information provided by transmitted light examinations, a short summary of the investigations using an optical microscope is given at the end of that subsection. The fourth subchapter describes microstructure investigations conducted in the scanning electron microscope. Due to test similar results conducted on thin sections and polished surfaces, the findings are presented in one subsection.

### 3.1. Accelerated Test of Mortar Expansion

**Figure 1:**
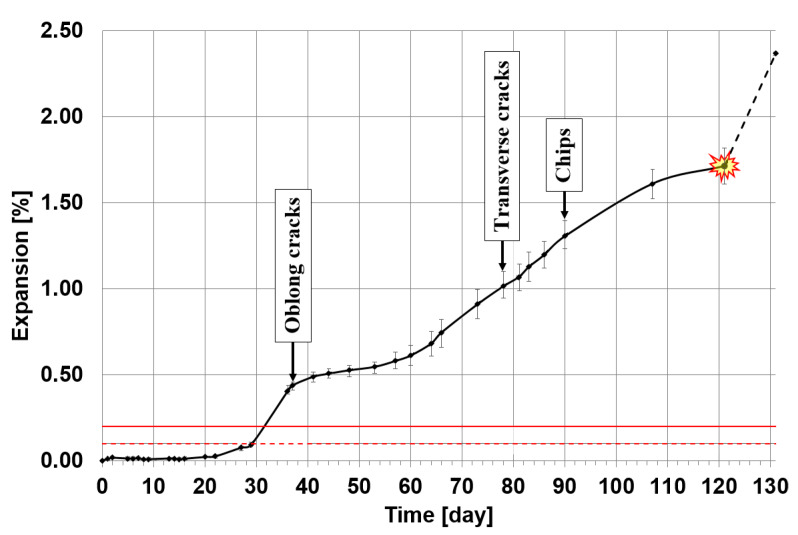
Expansion in the mortar with waste glass measured according to the ASTM C 1260 standard [38]. Measurement from 3 specimens until day 121 and measurement from 1 specimen conducted between day 121 and 131.

Figure 1 shows the progress of the expansion of mortar bars in which sand was replaced with waste glass.

**Figure 2:**
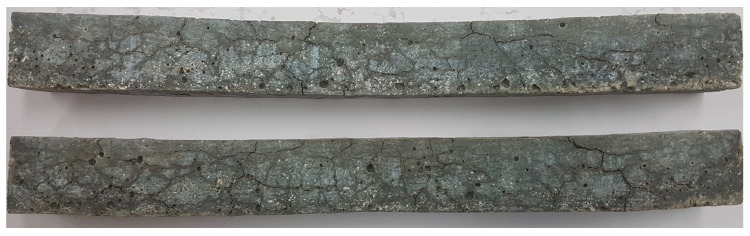
Cracked mortar specimens at 107 days of immersion in NaOH solution.

Despite the fact that waste glass is made of very reactive amorphous silica, in the early stage of the test period no significant expansion was observed as in [21,29]. Initially, the specimen containing waste glass did not show any significant dimensional changes, as reported in other studies [19,30]. In this study, this stable period lasted for as long as 22 days, well beyond the 14-day duration of the standard test after which the specimen could be considered as non-susceptible to ASR degradation. After 22 days of testing, the mortar began to expand progressively. Expansion proceeded slowly up to day 29, without exceeding the threshold of 0.1% at which the specimen could be considered to be potentially at risk from ASR. However, the rapid increase in expansion between 29 and 36 days showed that the tested mortar degraded due to ASR. A visible trace of this was the appearance of longitudinal cracks on the surface of the specimens observed on after 38 days. After that time the rate of expansion growth decreased and was relatively insignificant until 64 days. Since then the rate of expansion growth increased significantly, although it was not as high as between 29 and 38 days. Subsequently, a new damage indicating specimen degradation was observed (Figure 2). Transverse cracks appeared at 78 days, and spalling started at 90 days. Finally, after 131 days, two out of three specimens collapsed and no further measurements could be made. The results of the previous expansion measurement on all three specimens at 121 days was 1.71%. Over the next 10 days, the last specimen expanded further by 0.66%. These expansion results are higher than those reported in many other studies [18,20,21,22,27,29,30,36] conducted as per ASTM C 1260 [38] for a shorter period. This allows expecting more significant microstructural changes in the mortar under test.

The 121-day period of measurements preceding the specimen destruction is also almost twice as long as that in similar studies conducted by Shin et al. [6], in which a higher ultimate expansion value was achieved. The difference may result from a different type of glass that was the subject of the study. Sodium glass was used in the present studies and borosilicate glass was tested in [6]. This proves that borosilicate glass should be used for standard tests [5] as it allows achieving faster degradation of ASR specimens. It should be noted that more than 2% greater expansion was reported by Saccani et al. at 14 days of testing [26] for mortars with sodium glass from end-use fluorescent lamps.

### 3.2. Reflected Light Microscopy

**Figure 3:**
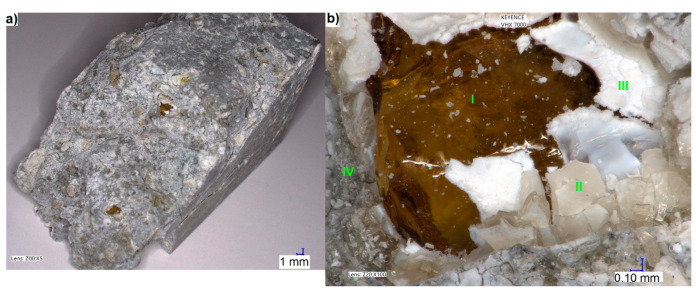
(**a**) Mortar bar with a natural crack surface; and (**b**) a degraded waste glass particle.

For microscopic examination, one of the mortar bars damaged by immersion in the 1M NaOH solution at 80 °C for 131 days was selected. First, without preparing any special test sections, the surface of the crack, responsible for breaking the continuity of the bar, was visually inspected (Figure 3). The exposed waste glass particles could be distinguished on the extensively cracked surface of the fractured bar (Figure 3a). Looking at it, one can notice that the core of unchanged brown glass (I) is surrounded by cracked, discoloured glass (II). Between the undamaged and cracked glass, there is a white alkali silica gel (III), an ASR product [52,54,55]. Cracked cement paste (IV) is observed around the glass particle.

**Figure 4:**
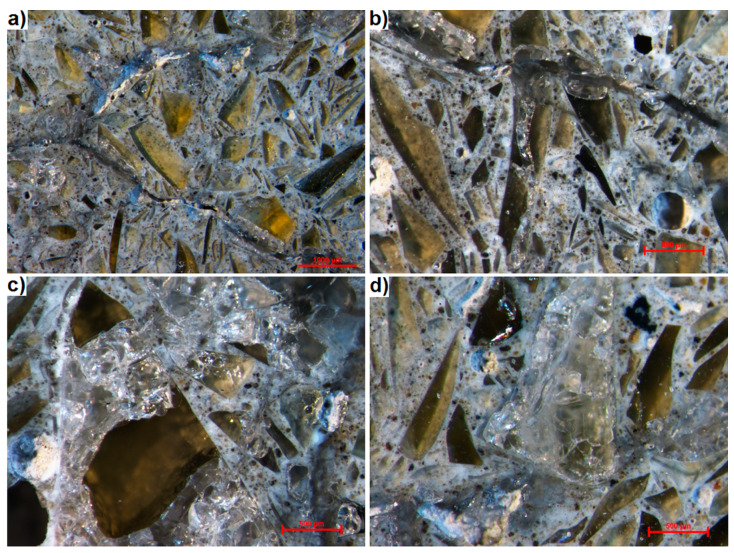
Polished section of mortar containing waste glass, obtained from reflected light microscopy, at (**a**) 20×; and (**b**), (**c**), (**d**) 40× magnification.

Figure 4 shows images of the polished section examined with optical microscopy in reflected light. These images show clearly yellow-brown, transparent glass particles embedded in the cement paste matrix. As in Szeląg et al. studies [55], no cracked region or a region with increased porosity is visible between the particles and the paste, which confirms a good bond between the particles and the paste. There is also no reaction rim around the glass grains that is sometimes observed around natural aggregates. [52,53]. The cracks of widths up to 0.36 mm are visible in the paste (Figure 4a), which is indicative of significant degradation of the mortar. They do not run along the glass-paste boundaries, but mainly throughout the paste. On the walls of these empty cracks, a white coating indicates the presence of alkali silica gel and other by-products (trona or thermonatrite) resulting from the immersion of the mortar in NaOH solution [56,57]. There are no alkali-silica reaction products in the air voids, as opposed to their very typical occurrence in this type of ASR concrete degradation [53].

Most glass particles show no signs of degradation. However, there are also those that clearly show the signs of degradation due to the contact with alkalis. The damage to these particles manifests itself in two ways. Some particles exhibit fractures when they stand in the way of the resulting crack (Figure 4b). Such a crack is not filled with anything, which may indicate that it is caused by an expansion processes taking place elsewhere in the sample. Discolouration of the glass particle is observed only in the direct vicinity of the crack. This type of degradation of glass aggregate is observed for small particles with sizes of less than 0.5 mm.

A different degradation type is observed in large glass particles and in some finer ones. Extensively cracked and discoloured regions are observed (Figure 4c), but the mechanisms of glass discolouration, which can vary, were not determined [58]. A network of small cracks with widths much smaller than the widths of cracks running throughout the paste is formed. A single glass particle may contain degraded and undamaged zones. Undamaged zones may also occur on the particle surface in direct contact with the cement paste. Thus, for such a particle, it is impossible to claim that the degradation starts on its surface and leaves an unreacted core when penetrating inward.

Extensively cracked glass particles larger than 1 mm, completely discoloured, are observed on the polished sections (Figure 4d) in the direct vicinity of large cracks propagating throughout the paste.

The described coarse crack propagation path may be considered typical of mortars subject to ASR degradation [52]. On the other hand, strong cracking of large glass grains is not typical, which may be explained by prolonged exposure to the alkaline solution in which the mortar was stored. The absence of the rim may suggest that ASR does not take place primarily on the aggregate surface, but in the cracks, as described by Maraghechi et al. [36]. This suggests that the mechanism of glass aggregate degradation should be considered according to the model proposed by Gao et al. [50].

### 3.3. Transmitted Light Microscopy

**Figure 5:**
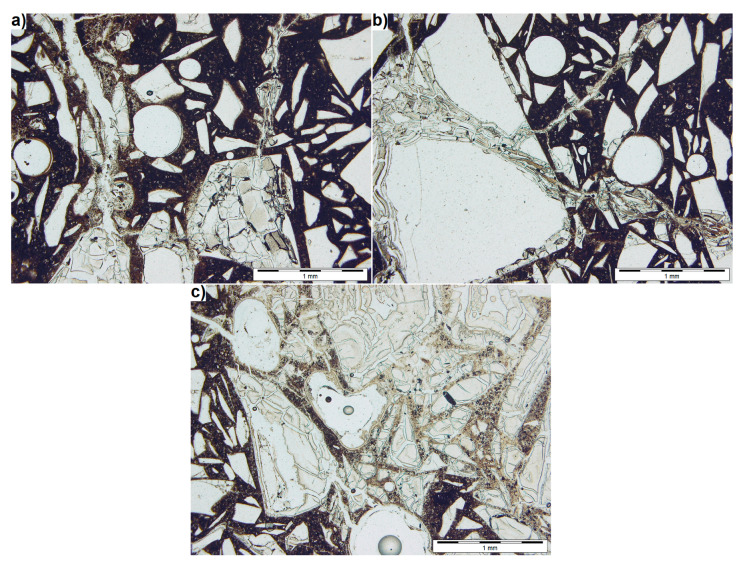
Thin section observed in transmitted light using a single polarizer. (**a**) typical area of cracked mortar with waste glass, (**b**) large waste glass grain having a single fracture, (**c**) strongly degraded mortar area.

Figure 5 shows the images of thin section obtained from transmitted light microscopy examination using one polarizer. Light-coloured glass particles and spherical pores are suspended in the dark cement matrix. As found in previous studies, glass particles adhere well to the paste but some of them being extensively cracked. This is particularly true for the particles with sizes exceeding 1 mm. Their whole surface may be degraded (Figure 5a), or the degradation of the particle may spread only along certain cracks formed in the particle (Figure 5b).

This technique allows distinguishing between the cracks and cement matrix. In Figure 5a,b, the cracks with widths up to 0.35 mm propagate from the large glass particles and run throughout the smaller particles, thereby degrading them. At the same time, the small particles located far away from the cracks show no signs of degradation. It is easier to recognize the propagation path of cracks in the mortar thereby making easier to confirm that the cracks propagate in the manner characteristic of ASR [52]. The difference is also significant in the case of testing degraded large glass grains, which in reflected light are more difficult to distinguish from the paste because of the discolouration.

In the immediate vicinity of the crack running through the paste, a discolouration of the paste can be observed (Figure 5a). It is more light-coloured, which can be explained by the formation of micro-cracks in the paste, increasing the degree of light transmission in this area. This also makes it possible to quickly identify regions where the ASR-induced degradation occurs to a greater extent. The image in Figure 5c is clearly brighter at the top, in the centre and on the right. In these regions, all the glass particles are cracked and the paste is highly transparent. At the bottom and left-hand side of Figure 5c, where more particles smaller than 0.5 mm are located, degradation occurs to a lesser extent. There are only single cracks in the glass and the paste is dark.

**Figure 6:**
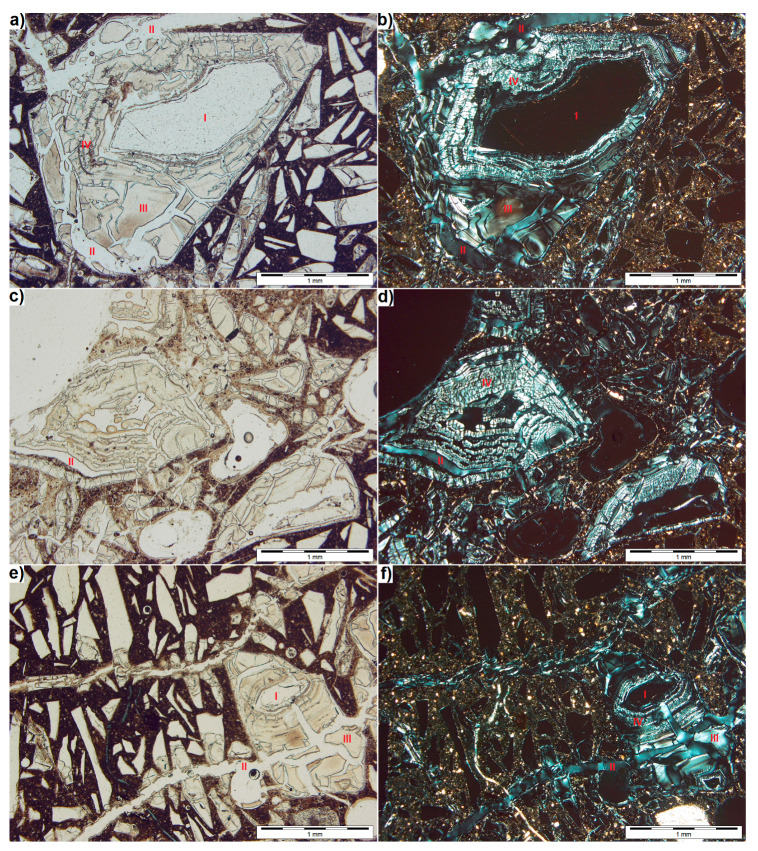
Thin section observed under transmitted light, obtained with one (left) and two polarizers (right); I – non-degraded glass, II – impregnating agent, III – degraded glass, IV – alkali silica gel. (**a**), (**b**) cracked large grain of waste glass; (**c**), (**d**) gel in the place of glass grain next to the air pore; (**e**), (**f**) cracks around the degraded glass grain.

Figure 6 compares images for the same regions obtained with transmitted light using one (Figure 6a,c,e) and two crossed polarizers (Figure 6b,d,f). The second polarizer allowed better identification of the composition of degraded mortar with waste glass and confirmed that many glass particles of size above 1 mm retained the core of undegraded glass (I). When only one polarizer is used, the colour of this core is similar to that of the impregnating agent used during thin section preparation. Examination with two polarizers reveals a distinct ‘opalescent’ colour of the agent (II). It can thus be concluded that all cracks are filled with the impregnating substance (Figure 6f), though it is not found in large air pores (Figure 6d).

As in Figure 3b, an area of degraded glass around the core can be distinguished, separated from the core by the alkali silica gel. When two polarizers are used, the degraded glass (III) looks similar to the impregnating substance, however, in contrast to it, it is a discontinuous phase. Also, when tested with one polarizer, the colour of degraded glass is darker than that of non-degraded glass and the impregnating agent. This is due to the lower light transmittance of such glass. In the test using non-polarized light, it is difficult to distinguish between ASR-degraded glass and the resulting gel. In Figure 6a only, one can see a rim of dendritic crystallites formed in the gel around the unreacted core. When two crossed polarizers are used, a typical microstructure (IV) of the gel is visible making it easily distinguishable from the glass. It can be seen that the gel inside the glass forms multiple layers up to 0.13 mm thick around the unreacted core (Figure 6b).

On the left-hand side of Figure 6d, there is a large particle of waste glass that turned into gel almost completely. In contrast to other degraded particles (Figure 6b,f), no degraded glass that may derive from alkali-silica reaction was observed around this particle, likely due to its location next to the large air pore visible in the upper left corner of Figure 6c. A large amount of NaOH solution could potentially accumulate in it, which would affect the ASR rate. The significant degree of degradation is also evidenced by the light-coloured paste observed in non-polarized light (Figure 6c). This indicates that the zone of more degraded mortar around the air pore is 2.3 mm deep.

Observations of the undegraded glass particles smaller than 0.5 mm revealed discontinuous, thin, bright rims (less than 0.01 mm wide) around them (Figure 6f). Their formation may be caused both, by the pores of the aggregate-paste interface being filled with the impregnate and by the formation of ASR products. This can be determined using the SEM analysis.

Analysis of the waste glass particles damage suggests that the degradation proceeds in two ways. Particles larger than 1 mm are more susceptible to ASR. This observation agrees with the results of the microscopic analyses (SEM) conducted by Idir et al. [19] and with the findings from the expansion tests for mortars with glass aggregate, for which the expansion is the greatest when the glass grain dimension is more than 1 mm [20,28,36].

As a result of ASR, the outer layer of glass is discoloured and cracked as the expanding alkali silica gel is formed underneath (Figure 6) [2]. Glass particle degradation is similar to that of opal aggregate, built exclusively of amorphous phase [40]. As the silica gel swelling, not only the outer layer of the glass is damaged but the resultant cracks propagate to the paste. Spreading in the paste, the cracks may encounter other glass particles on their way and cause their cracking. These cracks are the pathway that leads the hydroxide solution directly to the cracked aggregate. Subsequently, the alkali-silica reaction is initiated from the site where the crack is formed (Figure 4b, Figure 5b and Figure 6e,f). Thus, the process can be described as secondary degradation of glass aggregate due to ASR. The process occurs mainly in the particles smaller than 0.5 mm, which are less susceptible to ASR-induced degradation than the larger particles. This is consistent with the findings of Lee et al. [27] who found that the reduction of glass particle size below 0.6 mm may result in the reduced ASR expansion. At the same time, it is impossible to confirm that the finer fractions of the waste glass entered the pozzolanic reaction. The mortars were made to ASTM C 1260 [38] without glass aggregate grains less than 150 μm and thus it is safe to say that glass grain having pozzolanic properties were not used [20,25,26].

### 3.4. Scanning Electron Microscopy

**Figure 7:**
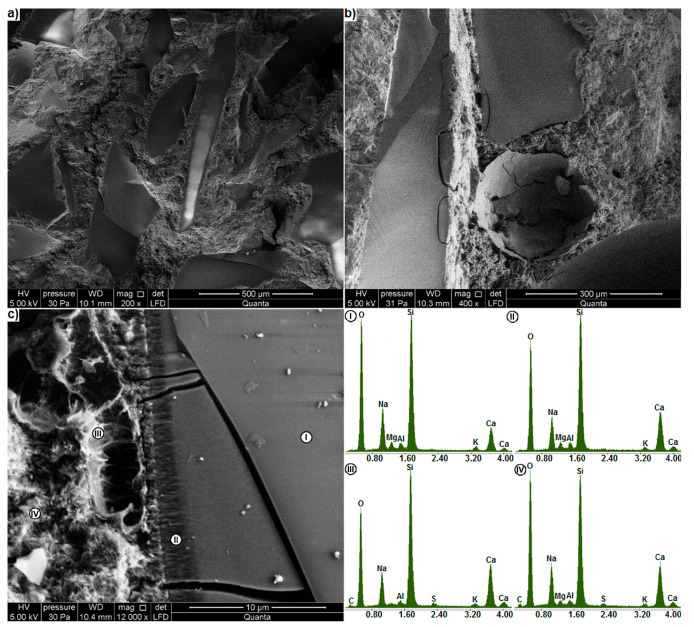
Mortar microstructure after 14 days of ASTM C 1260 test (SE). (**a**) General view, (**b**) cracked glass particles, (**c**) changes on the cracked glass particle surfaces, with EDS analysis in points I, II, III and IV.

Figure 7 shows the microstructure of the glass mortar after 14-day exposure in the NaOH bath at 80 °C. The test results confirm that within the ASTM C 1260 test duration [38], the alkali-mortar reaction does not cause any considerable changes in the mortar structure. No cracks are found in the mortar microstructure after 14 days of exposure to NaOH, and the glass particles adhere well to the cement matrix (Figure 7a). Single glass particles with minor cracks on their edges were detected (Figure 7b), mainly in the immediate vicinity of air pores. These can be residual cracks formed during glass grinding [36]. In their immediate vicinity no evident changes in the glass microstructure can be seen, unlike directly at its surface, which can be associated with a small width of the residual cracks, which is less than 2 μm.

The interface between such a particle and the paste observed at higher magnification showed other changes in the glass morphology, which may be attributed to the onset of ASR. A 2.75 μm thick fibrous gel layer can form in the glass near the surface (Figure 7c). Similar amorphous fibres at the edges of the aggregate particles were identified as ASR products in gneiss by Boehm-Courjault et al. [42]. The fibres in that layer were oriented perpendicularly to the surface and calcium in so altered glass gave more intense readings (II) than in the unaltered glass (I). A thin layer of gel products with a thickness of up to 1 μm formed also on the glass surface. Voids with single fibres of gel phase (III) can be seen between this layer and the cement paste (IV). For the phases occurring outside the glass particle ((I) and (IV)), the presence of sulphur was detected, which is related to ettringite produced during cement hydration [2].

**Figure 8:**
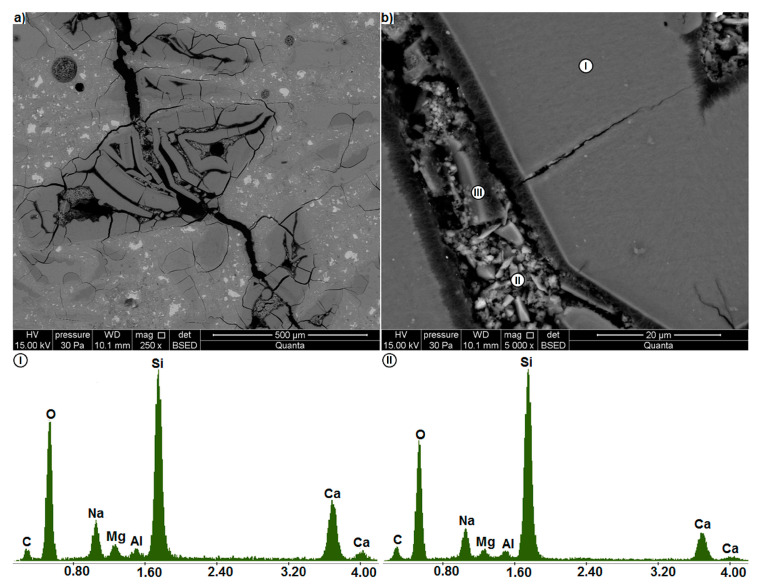
(**a**) Degraded mortar containing cracked waste glass particles, (**b**) a crack in the glass (BSE); EDS analysis at points I and II.

**Figure 9:**
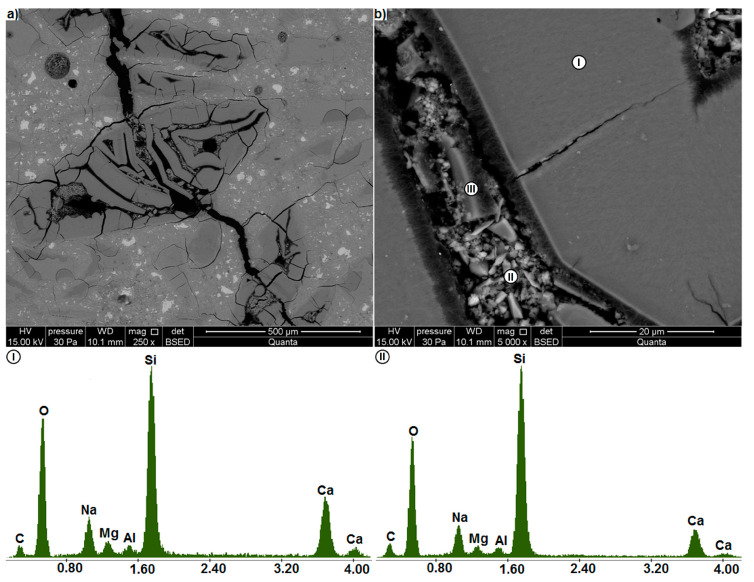
(**a**) extensively cracked region in the microstructure of the mortar containing waste glass (**b**) region of porous gel (BSE); EDS analysis at points I and II, (**c**) cracks in the gel.

**Figure 10:**
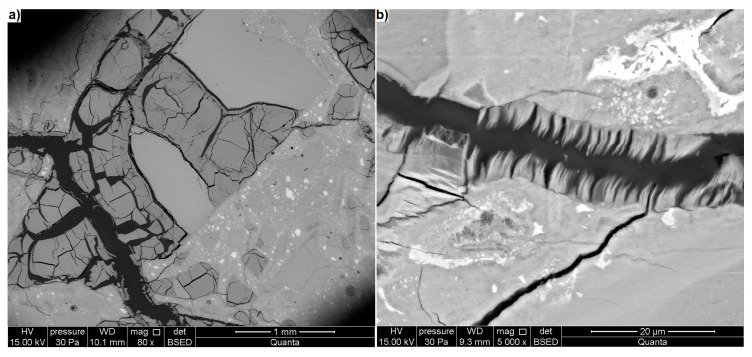
(**a**) large glass particle with a variable degradation level, (**b**) a crack running through the cement paste (BSE).

Figure 8, Figure 9 and Figure 10 show the microstructure of the glass mortar specimen after 131 days of exposure to 1 M of the 80 °C NaOH solution. Significant changes took place in the mortar microstructure between 14 and 131 days of the test. Just as optical microscopy, the SEM analysis showed the formation of a dense network of cracks. Figure 8 shows a crack that runs through the mortar and passes through glass particles smaller than 0.5 mm. The densely cracked, glass particles contribute to the degradation of the mortar. The mortar itself, however, is cracked only in the immediate vicinity of degraded glass grains and to a relatively small extent compared to the studies of Shin et al. [6] or Idir et al. [19].

The cracks in the glass may be empty the spaces or may be filled with crushed material (II) (Figure 8b). A 4.7 μm layer of gel was formed on the cracked surface of the glass. This gel is not as compact as the gel observed by Almesfer and Ingahm [51] or Shin et al. [6] during their studies on type 2 cracks. Inside the cracks, the broken fragments of the top layer of glass together with the gel were observed (III). This may suggest that the layer is not durable and, with the progress of ASR, it may deteriorate and be replaced by another, identical layer. Analysis of the elemental composition did not reveal significant differences between the glass composition (I) and the composition of the material filling the cracks (II). In the crack filling material, only the concentration of calcium is lower.

Figure 9a shows a densely cracked region in the mortar. The cracks propagate predominantly through the glass particles. Between them, there is a region of gel products of higher porosity (II) (Figure 9b). In this area, high silicon content was found; this can be attributed either to the glass particles proximity or to the gel being formed as a result of the waste glass reaction. The cracks spreading in the gel (I) were sealed by the products formed in them (Figure 9c). In addition to other elements present in that area, chlorine was also detected in the cracks. The microstructure of the examined gel resembles that observed on the shist particle in the studies of Leemann and Holzer [47]. Contrary to their findings, in the present study gel concentrations in mortar rarely occurred outside the cracked aggregate. Due to the limited area they occupy, they can be assumed to have formed at the sites of completely transformed aggregate grain. In the samples tested, there were no areas of cracked, compact alkali gel that often forms layers around the aggregate grains and fills the air pores in the mortars in which alkali-silica reaction occurs [7,8,19,20,29,53].

As shown in Figure 9a, glass particles of different colour shades were found. In BSE analysis, brighter spots are obtained where nuclei of elements with high atomic number are present. In the examined mortar microstructure, no crack propagation was observed in the light-coloured glass. Cracks propagated predominantly in the darker glass. This was particularly evident for glass particles larger than 1 mm (Figure 10a) and led to the conclusion that the degradation of darker glass particles was due to ASR. The darker colour of the glass can be related to linking atoms of light elements, sodium, oxygen and water [49]. At the same time, no trace of local dissolution of reactive particles was detected [7,41,47].

On the walls of cracks spreading through the paste (Figure 9c and Figure 10b), as in the cracks in the glass, the fibrous ASR products are visible (Figure 8b). Their appearance differed depending on the location. In the cracked glass particles, the fibres formed a relatively compact layer on the glass surface. In the paste, individual fibres up to 5 μm in length formed brush-shaped clusters.

**Figure 11:**
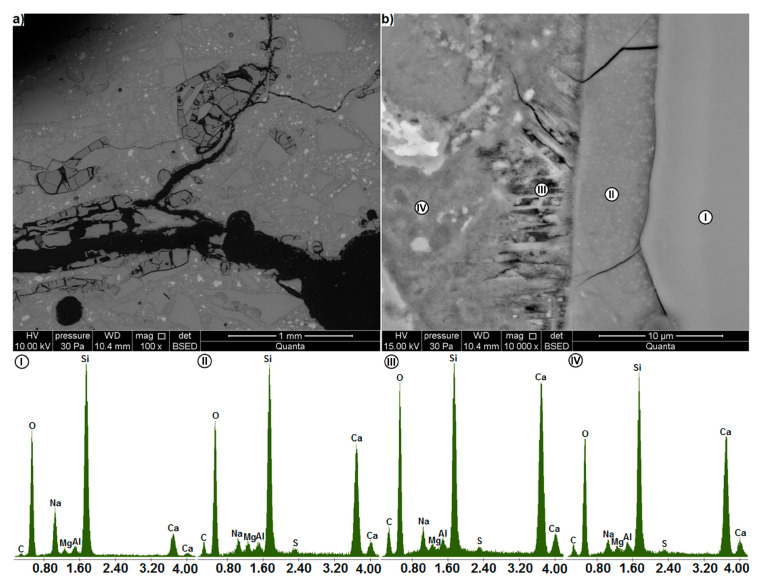
Microstructure of the mortar containing waste glass as examined on a thin section (BSE) (**a**) area around a large crack, (**b**) glass-paste interfacial zone; EDS analysis at points I, II, III and IV.

**Figure 12:**
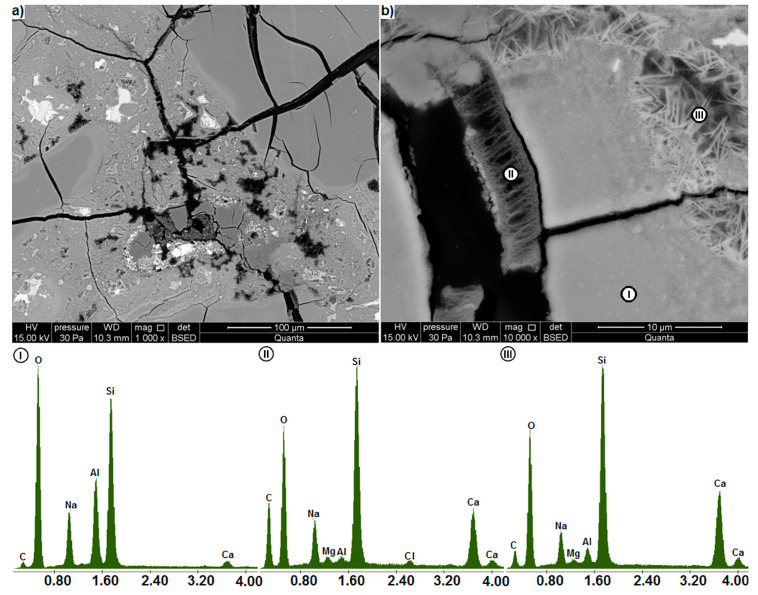
(**a**) extensively cracked area in the microstructure of the glass-containing mortar, (**b**) damage to waste glass particles (BSE); EDS analysis at points I, II and III.

Figure 11 and Figure 12 illustrate the images of thin sections of the specimens showing the microstructure of the mortar with waste glass. The microstructure observed on the thin section is similar to that on the polished section. Figure 11a shows a large 0.3 mm wide crack spreading through the mortar. In its immediate vicinity there are large, extensively cracked glass particles. In deeper parts of the paste there are smaller, undamaged or slightly cracked glass particles. Only single cracks run through the cement paste, which in the glass particles larger than 0.5 mm develop into a dense network of cracks. The glass-paste interfacial zone is typically only slightly damaged. A thorough visual inspection (Figure 11b) shows that undamaged glass (I) is the phase for which sodium shows relatively high concentration and simultaneously calcium - the lowest. In the surface layer of the glass (II) with single cracks, the intensity of the sodium peak decreases significantly and the intensity of the calcium peak increases. The results obtained are similar to those for the C-S-H phase (IV) surrounding the glass particles. This shows that the waste glass transformation occurs in the cement paste.

At the same time, an increase in the carbon peak was found (Figure 11). Carbon gives the most intense readings from the paste region near the surface of glass particles, which has the highest porosity (III). Differences in the intensity of carbon peaks can be attributed to the differences in the number of voids in the studied area. This is because they are filled with the carbon containing a polymeric impregnating substance. The carbon content increases by filling both cracks in the glass and the pores in the cement paste. The increased calcium content at the glass-paste interface, together with the plates arranged perpendicularly to the surface of the particles, indicate the presence of portlandite. This is a typical structure of the paste-aggregate interfacial zone with increased porosity in relation to the cement matrix [2].

As in Figure 9, a densely cracked area of mortar containing waste glass is visible in Figure 12a. There is also an area of porous gel between the glass particles. In the cracks running through the glass particles (Figure 12b) there are cracked layers of gel, up to 4.1 μm thick, similar to those in Figure 8b. However, only one such layer can be seen here, which, cracking transversely, splits between the two sides of the crack. A similar cracking mode was observed for the gel layers in nanocracks of reactive gneisses [42]. The thin cracks between the gel layer and the glass to which it adheres indicate its separation from the glass. In Almesfer and Ingham [51] microstructural studies of glass waste it is also visible. This is important because it shows that after a certain period of expansive gel formation, the gel layer disrupts and allows the alkaline solution to flow into the still unreacted glass. This mechanism can be used to explain the presence of crushed material observed in the cracks in Figure 8b and Figure 9a.

As in Figure 11b the EDS analysis in Figure 12b indicates that with the increased presence of voids in the region under examination, the intensity of signals from carbon increases. The strongest peaks are observed for the porous gel layer in the crack (II) and the weakest for the homogeneous glass (I). Around the glass, there is also a calcium-rich, porous layer filled with needle-like products (III), which may be portlandite and ettringite [2].

## 4. Conclusions

The performed tests allowed to characterize how the mortar with waste glass aggregate is degraded due to the influence of sodium hydroxide solution. For this purpose, various microscopic techniques could be used to obtain different information on the degraded mortar. The main conclusions were derived from this study are:

- the mortar with waste glass as aggregate shows little to no evidence of ASR within the standard test period, but at longer ageing times, the mortar can deleteriously expand until failure at 121 days;
- mortar expansion in mainly attributed to the presence of waste glass particles larger than 1 mm. The damage to glass particles can be easily determined during the preliminary visual test of fracture surfaces at very low magnifications;
- as a result of the reaction between large glass particles and alkalis, expansive alkali gel forms in the glass cracks. While propagating from the glass into the paste, the largest cracks up to 0.35 mm in width can damage other glass particles, exposing them to ASR damage;
- brown waste glass subjected to ASR becomes cracked, discoloured and less transparent;
- unreacted glass is most easily distinguished by optical microscopy in reflected light, due to the ASR-induced glass discolouration. The discoloured glass is similar in colour to the surrounding cement matrix. This is because of its transparency, although due to the cracks and transformations, the glass becomes less transparent than unreacted glass, as evidenced by microscopic examination in non-polarized transmitted light;
- non-polarized light examination allows easy identification and measurement of the cracks propagating in the mortar. Increased transparency of the cracked regions enables the examination of the damaged paste areas in the vicinity of reactive aggregates and cracks;
- two crossed polarizers should be used for the examination in transmitted light to identify the gel phase forming as a result of ASR;
- high magnifications in SEM allow early detection of ASR symptoms and examination of the phases that fill the cracks;
- SEM images of polished and thin sections are similar, but owing to the impregnating agent that fills the cracks and pores (except air voids) in the thin section, additional qualitative interpretation of the porosity of the examined region is possible based on the carbon peaks intensity. The polymer resin used for the impregnation is the only carrier of carbon when no evidence of specimen carbonation is found;
- given the considerable expansion of the specimens in the accelerated test, further research into the use of brown waste glass as aggregate should check whether the mortar exhibits the symptoms of ASR-induced degradation in the prolonged long-term testing (e.g., ASTM C227 [59]).

## Figures and Tables

**Table 1:** Chemical composition of cement and glass.

Chemical Composition	SiO	CaO	Al_2_O_3_	MgO	Fe_2_O_3_	Na_2_O	K_2_O	SO_3_	Cl
**Cement**	19.07	63.99	5.43	1.66	2.79	0.25	0.99	3.41	0.069
**Glass**	63.89	11.65	4.00	1.25	-	19.21	-	-	-
