# Correction: Czapik, P. Microstructure and Degradation of Mortar Containing Waste Glass Aggregate as Evaluated by Various Microscopic Techniques. *Materials* 2020, *13*, 2186

**DOI:** 10.3390/ma14010159

**Published:** 2020-12-31

**Authors:** Przemysław Czapik

**Affiliations:** Department of Building Engineering Technologies and Organization, Kielce University of Technology, Al. Tysiąclecia Państwa Polskiego 7, 25-314 Kielce, Poland; p.czapik@tu.kielce.pl

The author wishes to make the following correction to this paper [1]. Due to the duplication of the same diagrams in Figures 8 and 9, replace

**Figure 9:**
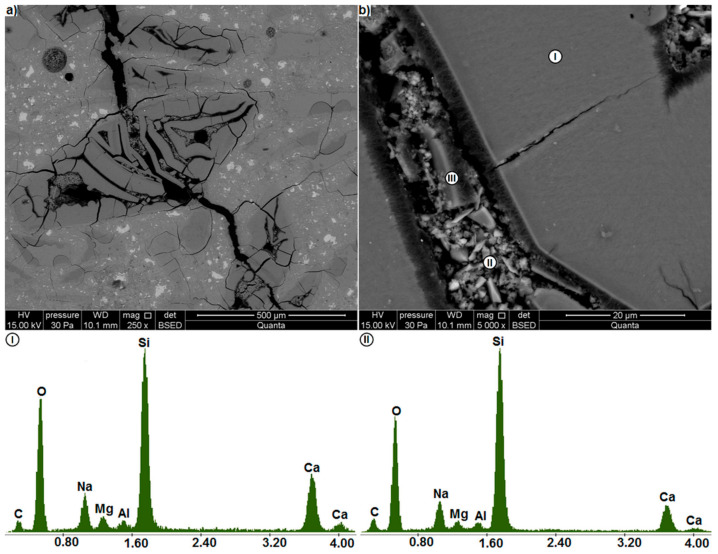
(**a**) extensively cracked region in the microstructure of the mortar containing waste glass (**b**) region of porous gel (BSE); EDS analysis at points I and II, (**c**) cracks in the gel.

with

**Figure 9:**
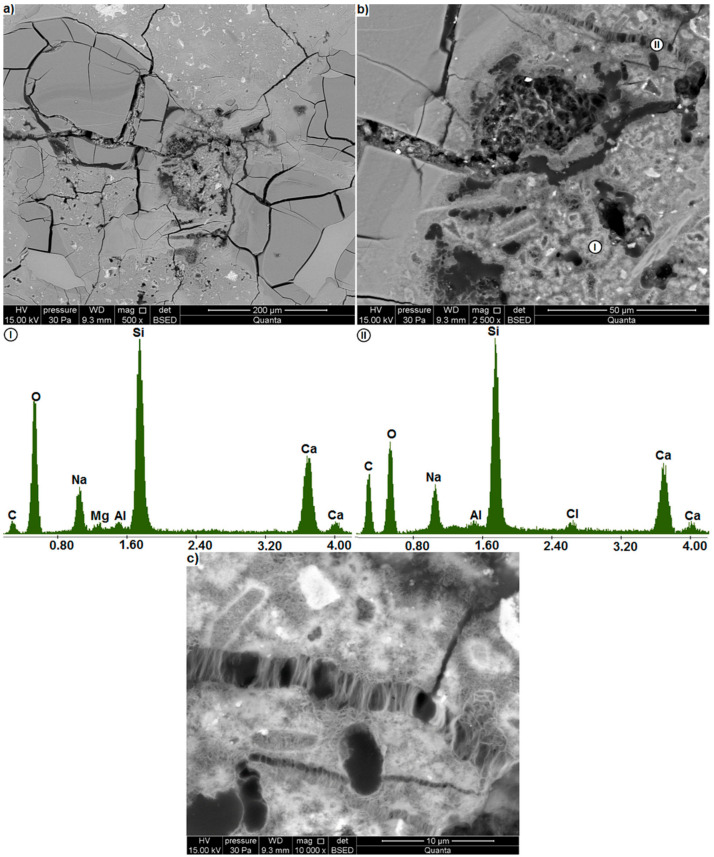
(**a**) extensively cracked region in the microstructure of the mortar containing waste glass (**b**) region of porous gel (BSE); EDS analysis at points I and II, (**c**) cracks in the gel.

The author would like to apologize for any inconvenience caused to the readers by these changes. This change will not affect the results of the article.
